# Heterotopic Ossification: A Challenging Complication of Total Hip Arthroplasty: Risk Factors, Diagnosis, Prophylaxis, and Treatment

**DOI:** 10.1155/2019/3860142

**Published:** 2019-04-16

**Authors:** Paweł Łęgosz, Maciej Otworowski, Aleksandra Sibilska, Krzysztof Starszak, Daniel Kotrych, Adam Kwapisz, Marek Synder

**Affiliations:** ^1^Department of Orthopaedics and Traumatology of the Musculoskeletal System, Infant Jesus Teaching Hospital, Medical University of Warsaw, Warsaw, Poland; ^2^Clinic of Orthopaedics and Paediatric Orthopaedics Medical University of Lodz, Lodz, Poland; ^3^Department of Orthopaedics, Traumatology and Orthopaedic Oncology, Pomeranian Medical University of Szczecin, Pomeranian Medical Academy Oncology Therapy and Research Center, Szczecin, Poland

## Abstract

**Background:**

This review is intended to summarize the risk factors, classification, diagnosis, and treatment of heterotopic ossification (HO) of previously published studies.

**Results:**

Heterotopic ossification is a common complication of total hip arthroplasty. Its prevalence is not the same in all of the patient groups. Frequency of HO varies from 15 to 90%. Hip ankylosis, male gender, and previous history of HO are said to be risk factors with a significant level. Diagnosis is based on a single AP radiograph: the Brooker classification that divides HO into four grades is the most commonly used. The confirmation test that can be used is a bone scan. A great amount of bone metabolic turnover markers have been tested, but none of them seems to be relevant in case of prevention or diagnosis of HO. The most effective prophylactic treatment is radiotherapy or administration of nonsteroidal anti-inflammatory drugs. Over the years a lot of different RT protocols have been tested. Nowadays the most often used regimen is 7 Gy given postoperatively in a single dose. The most commonly prescribed drug in prophylaxis of HO is indomethacin. Also, the efficacy of ibuprofen and diclofenac was proven. Recently researchers focused on selective COX-2 inhibitors. They appear to be as effective as nonselective NSAIDs having less side effects. The one and only treatment of HO is a revision arthroplasty.

## 1. Introduction

**Figure 1:**
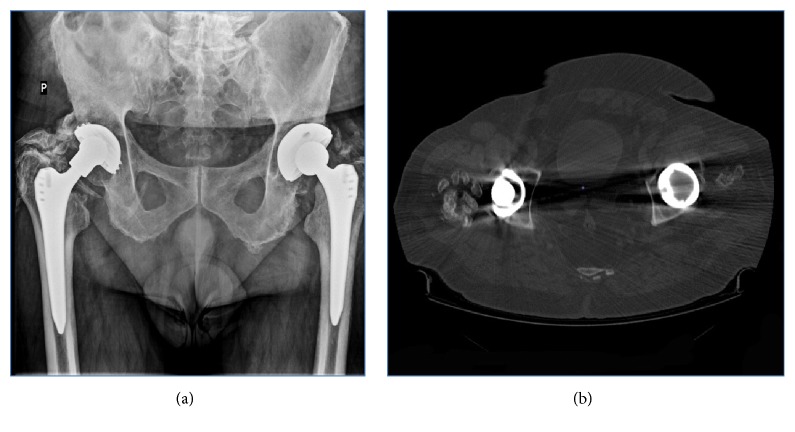
Heterotopic ossification visible on an AP X-ray (a) and on a computed tomography scan (b) around right hip. It is a complete hip ankylosis. HO like this can completely sabotage functional outcomes of THR limiting the range of motion in all planes. This is why using right prophylaxis is so crucial (from the Department of Orthopaedics and Traumatology of the Musculoskeletal System, Infant Jesus Teaching Hospital, Medical University of Warsaw records).

Heterotopic ossification (HO) (Figure 1) is a relatively common complication of hip arthroplasty procedures. According to data from several studies, their frequency varies from 15 to 90% depending on the investigated group of patients [1–13]. The latest meta-analysis from 2015 presented the average frequency of their occurrence at the level of 30% [14]. Considering the epidemiology of hip arthroplasty ossifications affect a significant percentage of patients. These figures are likely to steadily increase until the development of specific treatment strategies. None of the numerous international or national orthopaedic associations have yet developed guidelines for the prevention and management of patients with existing heterotopic ossifications.

This is an extremely important challenge, as ossifications may destroy the healing effect of surgery, limiting to a large extent of range of motions in the hip joint and additionally exposing the patient to subsequent surgical treatment, revision arthroplasty.

## 2. Methodology

A search was performed on PubMed, Embase, and Cochrane Central Register. It was conducted using key word “heterotopic ossification” combined with either “THR”, “total hip replacement”, “THA”, “total hip arthroplasty”, “NSAIDs”, “risk factors”, “radiotherapy”, “radiation”, “marker”, or “markers”. Also, the search of references in included studies was performed. There were no restrictions in language or date of publication. Studies were excluded from our analysis if there were abstracts, letters, case reports, case series, guidelines, or irrelevant significance for our study topic. We identified 4628 articles. Of these articles, 836 were removed due to duplicate reportage, and 3675 were excluded based on the titles and abstracts. The remaining 117 articles were accessed for the full text and screened for further assessment. Finally, we included 63 articles in our review.

## 3. Risk Factors

A number of studies demonstrating the existence of potential risk factors for heterotopic ossifications after hip arthroplasty have been found in the literature [3, 4, 19–24].

In the recent meta-analysis it was estimated that male sex, cemented prosthesis, bilateral hip joint arthroplasty procedure, ankylosing spondylitis, and hip joint ankylosis are associated with increased risk of nonarticular ossification [14]. In addition, the above factors have been divided into those with significant and moderate level of risk of HO. The first group included hip ankylosis and male gender. The group with a moderate level included cemented type of prosthesis, bilateral procedure, and ankylosing spondylitis.

According to the same study, the only factor that is said to decrease HO is rheumatoid arthritis [Table 2].

In addition, it has been demonstrated that surgeries due to femoral neck fracture, degenerative disease, previous hip fracture, hypertrophic type of osteoarthritis, and age are not associated with an increased risk of heterotopic ossification.

There have been studies evaluating the assessment of the impact of revision surgery, lateral approach in hip arthroplasty, BMI, bone grafting, and trochanteric osteotomy on the potential occurrence of ossification but even if they reported a positive correlation they require further research [14].

## 4. Classification

The most commonly used method of classification is Brooker classification based only on a single X-ray image in the anteroposterior (AP) projection [21, 25, 26].

Usually, ossifications that develop after surgery are visible in the radiological examination after 4-6 weeks [2, 5, 6, 8, 12, 13, 27–30].

**Figure 2:**
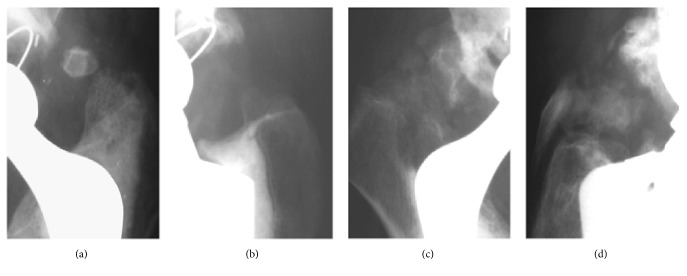
The presence of HO Graded in Brooker Classification as I (a), II (b), III (c), and IV (d) on a follow-up after THR (from the Clinic of Orthopaedics and Paediatric Orthopaedics Medical University of Lodz records).

The Brooker classification divides the heterotopic ossifications into four grades (Figure 2):

I - described as islands of bone within the soft tissues about the hip;

II - consisting of bone spurs originating from the pelvis or proximal end of the femur, leaving at least 1 cm between opposing bone surfaces;

III - consisting of bone spurs originating from the pelvis or proximal end of the femur, reducing the space between opposing bone surfaces to less than 1 cm;

IV - hip joint ankylosis [25, 26].

Types III and IV are referred to as clinically relevant [12, 21, 30, 31].

## 5. Diagnosis and Decision Making

The level of alkaline phosphatase in serum increases during tissue injuries; therefore its predictive value in heterotopic ossifications after hip arthroplasty is doubtful [32]. However, it is still used in some clinical conditions [32]. Over the years, many attempts have been made to determine alkaline phosphatase in serum but it seems that this method is not specific without clear evidence for its predictive value. Both Mollan and Kjaersgaard did not report any significant correlation when assessing the dependence of alkaline phosphatase levels on the occurrence of heterotopic ossifications [33–36].

Wilkinson et al. tried to find alternative bone metabolic turnover markers that could be used in the diagnosis of heterotopic ossifications in patients after hip arthroplasty. According to their study, C telopeptide of type I-collagen (CTX-1) is potentially useful marker, which increases its concentration in the first postoperative week by 42% and shows a sensitivity of 89% and specificity of 82% in the prediction of the diagnosis of ossifications in the 26th week after surgery [33]. Unfortunately, such findings are not sufficient to assess the validity of routine CTX-1 measurements, as indomethacin therapy should be implemented on the first postoperative day, and radiotherapy is most effective if it is performed on the fifth postoperative day [7, 17, 28, 37, 38].

Based on Wilkinson's research, we can reject N-terminal propeptide of type I-procollagen (PINP) and osteocalcin (OC) from potential testings, as their increased correlation with HO occur too late to implement effective prophylaxis. Moreover, the levels of N-telopeptide of type I-collagen (NTX-1) and deoxypyridinoline (DPD) were not correlated at all with the presence of ossifications [33].

Bone metabolic turnover markers have also general diagnostic limitations. Their level fluctuates in relation to gender, coexisting diseases and some are prone to daily or seasonal fluctuations, which makes them difficult to use as a simple diagnostic tool [33].

In papers heterotopic ossification symptoms include swelling, redness, and pain as subjective symptoms. Therefore, symptoms of ossification may imitate deep vein thrombosis in the lower limbs or cellulitis. Unfortunately, none of these studies are supported by the relevant figures [12, 32, 36, 39–41].

Scintigraphy is the most sensitive method in diagnosing HO [32, 36, 42]. Changes in the X-ray images may be preceded by the changes in scintigraphy with a time of 4 to 6 weeks [41].

The formerly used radiopharmaceutical was strontium, currently 99mTc; pyrophosphate is used [22, 41, 42]. Pyrophosphate is a calcium metabolism regulator; it accumulates in places where the metabolic turnover of calcium is increased [22].

Scintigraphy is based on the administration of a radiopharmaceutical and the subsequent production of a series of scans that will provide information during three phases: a dynamic blood flow, blood accumulation, and the accumulation of a radiopharmaceutical in the bone [41].

Scintigraphy is particularly effective in determining the maturity of heterotopic ossifications and planning their operational removal [39, 42]. The maturity of ossification is indicated by a decrease in radiopharmaceutical uptake when comparing the results of subsequent scintigraphic tests [39].

Schurch et al. conducted a study to evaluate the usefulness of prostaglandin E2 (PGE-2) measurements in urine to predict the formation of HO. They reported increased level of PGE-2 in urine in patients with developing ossification. The authors recommended examining 24-hour PGE-2 level once a week for a period of 3 to 4 months. The increase in this excretion was expected to indicate the development of heterotopic ossification [40].

Orzel et al., in their retrospective analysis of 24 patients' ossifications, showed that calcium levels in serum had decreased below the reference level in 23 cases. The reduction occurred on average during fifth day and lasted on average ten days [36]. It seems that it would not be clinically useful to use this as a diagnostic test either, due to the late outcomes compared to the optimal time after surgery to undertake prophylaxis.

*Prophylaxis of Heterotopic Ossification: Methods and Their Effectiveness*

*(1) Radiotherapy (RT). *It is estimated that the prevalence of HO should not be greater than 10% after the radiation prophylactic treatment [43, 44].

Over the years many RT regimens have been tested. The first observation was published by a team from Mayo Clinic. They used fractionated RT (2 x 10 Gy) [29]. Then researchers tried to find the lowest effective dose [5, 17, 18, 28, 37, 45–47].

In a meta-analysis Milakovic et al. found no significant differences in a HO prevalence between the patients treated with banana equivalent dose (BED)>2500 cGy and the patients treated with BED doses <2500 cGy [2]. Healy et al. reported that radiating the hip with a single dose of 550 cGy is less effective than with a 700 cGy [37].

The difference in HO occurrence was so significant that the researchers advised not to use that protocol again. It seems that Finegorth et al. found the boundary of an effective radiation protocol with a 6 Gy given postoperatively [17].

Nowadays the most often prescribed is a single dose radiation of 700 Gy [2].

Also, the effectiveness of preoperative radiation has been measured [3, 19, 27, 43]. Radiation prescribed before the procedure limits the patient discomfort and reduces the risk of hip dislocation [3, 19, 48]. Gregoritch et al. concluded that there is no difference between the treatment given 6 hours before the procedure and the treatment given 72 hours postoperatively [48]. Milakovic and Seegenschmiedt in a large, multicenter study showed no differences between radiation prescribed before the operation and after the operation [2, 18]. However, it appears that there is less indications for preoperative radiation than for postoperative radiation. The preoperative regimens are ineffective if the patient is operated on HO graded as III or IV in Brooker classification [2, 18].

The most important factor in a prophylactic radiation treatment is time. Seegenschmiedt concluded that it must not be given earlier than 8 hours before the procedure and later than 72 hours after the procedure [18]. The other authors are in agreement that hip should be radiated within 5 days after the operation [5, 6, 28, 38, 44, 45, 49].

Finally, the fractionated radiation has been compared with a single dose radiation. Konsi et al. in a randomized study showed the same efficacy of a single dose radiation compared to a fractioned one [38]. Milakovic et al. concluded that a single dose radiation was associated with a higher prevalence of HO, but only in lower grades of Brooker classification [2].

RT is not only effective, but safe. Sheybani et al. analyzed records of over 3500 patients who underwent a THR [50]. They concluded that there is no elevation of the malignancy risk after the radiation prophylaxis treatment.

*(2) Relevance of Using Medications*

*(a) Nonsteroidal Anti-Inflammatory Drugs (NSAIDs). *The most commonly used drug in HO prophylaxis is indomethacin. It is probable mechanism of stopping the bone formation is by inhibiting the inflammation process [4].

Schmidt et al. in a prospective, double-blind, randomized study proved the effectiveness of indomethacin in HO prevention [15]. It was given 3 times a day in a 25 mg dose for 6 weeks after the procedure. No ossification was graded greater than I in Brooker classification. Kjaersgaard in his study used the same dose as Schmidt, but prescribed it for 2 weeks [51]. It was as effective as the 6-week therapy.

Another drug whose efficacy has been proved is ibuprofen. Elmsted et al. showed its effectiveness compared to a placebo [52]. Sodemanna et al. showed no difference in HO occurrence between the group treated with indomethacin (50 mg/d) and the group treated with ibuprofen (3 x 400 mg/d) for the same time (20 days) [53].

It appears that the shortest possible treatment time with nonselective oral NSAIDs is one week [7]. It should not be started before the procedure, because ineffectiveness of such intervention has been proved and it elevates the risk of an excessive bleeding during the procedure [7].

Pritchett studied the efficacy of ketorolac in a prospective, double-blind, randomized study [54]. It was administered in injections. The protocol started during the arthroplasty and ended 48h after the procedure. None of the patients developed clinically significant HO.

It appears that there are no big differences between the two most studied prophylactic treatments. Kienapfel et al. conducted a study that compared indomethacin given in a dose of 2x 50 mg for 42 days with a single dose 600 Gy postoperative radiation. The treatments were equally effective in preventing HO [4].

Knells et al. conducted prospective study comparing indomethacin with acetylsalicylic acid and radiotherapy. They concluded that the most effective prophylactic treatment for the general population is indomethacin prescribed in a dose of 250 mg daily for 14 days starting on the first postoperative day. The patients with a higher chance of developing HO or with the contradictions for nonselective NSAIDs should be qualified for RT [55].

Kolbl et al. compared preoperative radiation (7 Gy 16-20h before the procedure) and diclofenac (2x75mg for 14 days starting at the 1st postoperative day). They showed no difference in lowering the clinically significant HO occurrence, although the overall prevalence of HO was lower in diclofenac group [3].

The only prospective study evaluating efficacy of combined therapy conducted Pakos et al. [56]. They compared combined prophylactic therapy (radiotherapy + indomethacin) with indomethacin alone. There were two prospective groups: patients older than 55 y.o. treated with a single dose 7 Gy radiation and indomethacin given in a dose of 75 mg for 15 days; patients younger than 55 y.o. treated with indomethacin given in the same dosage and continued for the same amount of time. To reduce the influence of age on the outcomes the historic group was created: it consisted of patients older than 55 y.o. treated with indomethacin in the same dosage continued for the same amount of time. HO was less frequent in the first group than in the other two (8% versus 27% versus 26%).

Selective cyclooxygenase-2 (COX-2) inhibitors are a very attractive form of treatment, because they show less gastrointestinal side effects and lack interaction with platelets aggregation [8, 9, 11, 12, 23, 57]. However, it should be mentioned that studies showed elevated cardiovascular risk during the treatment with a selective COX-2 inhibitors [8, 9, 11, 12, 58].

The elevated cardiovascular risk was observed after 6 months of treatment (also, 12 or even 18 months were mentioned in other studies), period way longer than prophylactic treatment time after total hip replacement (THR) [12, 13].

Rofecoxib and celecoxib has been the most studied agents and proved reliable [8].

Lavernia et al. proved effectiveness of etoricoxib compared to historical group that did not take any prophylaxis at all [9]. Patients received etoricoxib in a dosage of 200 mg twice daily for two weeks. Walther et al. reported HO 6 months after the procedure in 30% of the patients treated with a rofecoxib (2 out of 137 were graded as II in Brooker classification) [57]. At a dosage of 25mg continued for 2 weeks starting on the first or second postoperative day rofecoxib showed the effectiveness as a diclofenac described in a literature at a dosage of 100-150mg. The study conducted by Winkler et al. gave us even more information about etoricoxib. It was a prospective, double-blind, randomized study that compared etoricoxib (90 mg/d) and diclofenac (70 mg/d) both given for 9 days, starting the first day after the procedure [8]. No difference was found in HO occurrence.

Finally, the very conclusive results came with both Grohs and van der Heide studies [10, 58]. They compared rofecoxib with indomethacin. The rofecoxib group in Groh's study was given it in a dosage of 25 mg daily for 7 days and in van der Heide's 25 mg twice daily for 7 days. The indomethacin group was given it in a dosage of 50 mg twice daily in the first study and thrice in second. Grohs reported lower occurrence of HO in rofecoxib group. Also, he reported greater prevalence of HO graded as III or IV in Brooker classification. Van der Heide showed no significant difference between two groups in HO occurrence (87% versus 85%).

Oni et al. conducted prospective study with celecoxib [12]. Patients had taken it in a dosage of 200 mg daily for 6 weeks, starting at the first postoperative day. The control group consisted of patients that had not taken any prophylaxis. HO was less frequent and the Harris Hip Score (HHS) score was higher in the celecoxib group, although as much as 10 percent of the ossifications in this group were graded as III in Brooker classification. Romano compared celecoxib (2x200 mg) with indomethacin (2x500 mg); both were prescribed for 20 days starting the first postoperative day [23]. After the year, in the group treated with celecoxib they reported lower occurrence of HO (17,5% versus 14,3%); also in the celecoxib group there were less discontinuations due to gastrointestinal side effects (2% versus 8,4%).

Saudan et al. compared celecoxib (2x200 mg) and ibuprofen (3x400 mg). Celecoxib turned out to be more effective in preventing HO than ibuprofen [13].

Xue et al. conducted a meta-analysis of randomized studies that compared NSAIDs with selective COX-2 inhibitors [11]. They found no significant differences in overall HO prevalence and in clinically significant ossifications prevalence. Moreover, the patients treated with selective COX-2 inhibitors showed less discontinuations due to gastrointestinal side effects.

Another meta-analysis by Shun Lin et al. showed better outcomes in HO prevention after NSAIDs prophylaxis treatment compared with placebo. In this study, researchers also measured the discontinuation of the therapy due to gastrointestinal side effects and compared it between groups treated with nonselective NSAIDs and selective COX-2 inhibitors. The discontinuation was more frequent in the nonselective NSAIDs group.

*(b) Diphosphonates. *The prophylaxis of HO that was proven to be ineffective is treatment with diphosphonates [6, 20, 44, 46, 47, 49]. This therapy only delays development of HO. Diphosphonates inhibit hydroxyapatite crystals growth, but has no effect whatsoever on the production of the osteoid matrix [6, 20, 44, 47, 59]. After the therapy is ended previously created scaffold begins to calcify and therefore it could be visualized on radiographs [20, 44, 47, 59]. Thomas et al. compared two patients' groups that took diphosphonates starting 2-4 weeks before the operation and continuing it for 3 weeks after the procedure [20]. The first group took them in a dosage of 10 mg/kg, and the second in a dosage of 20 mg/kg. In both groups diphosphonates were equally ineffective in preventing HO.

Bijovet et al. also proved inefficacy of diphosphonates in HO prophylaxis by conducting a prospective study [59]. Patients started the therapy 6 weeks before the procedure and ended it 6 or 12 weeks after the procedure. The prescribed dose was 20 mg/kg given daily. On the radiographic follow-up during the treatment only 10% of the patients revealed HO. However, 2 to 3 months after the termination of the therapy HO was diagnosed in 60% of the patients.

## 6. Treatment of Manifested HO

Grades I and II HO may not need any treatment unless they become symptomatic or functionally disabling. The gold standard therapy for grades III and IV HO is a revision arthroplasty and surgical resection of the ossification [5, 6, 13, 32, 35, 36, 42, 46]. To lower the risk of intraoperative complications such as excessive bleeding and postoperative complications such as recurrence the procedure must be performed when the HO is mature [32, 42]. Also, it should be kept in mind that the strongest risk factor of HO development is the previous history of HO, so prophylaxis treatment must be administered after the operation [14]. It has been proven that incorporating of appropriate care managers into health system who work directly with individual patients helps them with lifestyle changing, monitors their condition, and ultimately influences a better compliance with the suggested recommendations [60].

## 7. Conclusion

HO is one of the most common complications after THA. Until now, no algorithm for detection and treatment has been developed. A series of papers agree on risk factors and we collected those in Table 2. Many biochemical markers have been measured, but none of them have been widely used in clinical practice. Scintigraphy seems to be the most sensitive method in diagnosing but X rays are the most frequently used. As regards prophylaxis, the effects of radiotherapy (pre/postoperatively) and pharmacotherapy have been proven. NSAIDs and COX2 inhibitors are commonly used. HO is a problem for a large number of patients and further research should be performed. The most relevant findings in literature are gathered in Table 1.

## Figures and Tables

**Table 1:** Summary of the most relevant finding in literature.

Author	Year	Results
Schmidt et al. [15]	1988	Indomethacin is especially recommended for patients who are at high risk for HO.

Wright et al. [16]	1994	The severity of HO did not correlate with the HHS; the relationship between HO and range of motion (ROM) indicates that the Brooker index is a valid measurement.

Fingeroth et al. [17]	1995	A single dose of 6 Gy of radiation given within the first 3 postoperative days provides effective prophylaxis against HO.

Seegenschmiedt et al. [18]	2001	Both preoperative (within 24 h) and postoperative RT (within 72 h) are effective in preventing HO after hip surgery.

Saudan et al. [13]	2007	Celecoxib was more effective than ibuprofen in preventing heterotopic bone formation after total hip replacement.

Xu et al. [11]	2014	Considering the side effects of nonselective NSAIDs, selective COX-2 inhibitors were recommend for the prevention of HO after THA.

Lavernia et al. [9]	2014	A short course of celecoxib for pain aids in the prevention of HO after primary THR.

Winkler et al. [8]	2016	Etoricoxib and diclofenac are equally effective for oral HO prophylaxis after primary cementless THA when given for nine peri-operative days.

**Table 2:** Risk factors of heterotopic qssification.

Level of risk	Factor
High	Hip ankylosis, male gender, previous history of HO.

Medium	Cemented type of prosthesis, bilateral procedure, ankylosing spondylitis.

Low	Rheumatoid arthritis
